# Wharton’s jelly-derived mesenchymal stem cells combined with praziquantel as a potential therapy for *Schistosoma mansoni*-induced liver fibrosis

**DOI:** 10.1038/srep21005

**Published:** 2016-02-15

**Authors:** Olfat A. Hammam, Nagwa Elkhafif, Yasmeen M. Attia, Mohamed T. Mansour, Mohamed M. Elmazar, Rania M. Abdelsalam, Sanaa A. Kenawy, Aiman S. El-Khatib

**Affiliations:** 1 Department of Pathology, Theodor Bilharz Research Institute, Warrak El-Hadar, Imbaba, P.O. Box 30, Giza 12411, Egypt; 2 Department of Electron Microscopy, Theodor Bilharz Research Institute, Warrak El-Hadar, Imbaba, P.O. Box 30, Giza 12411, Egypt; 3 Department of Pharmacology, Faculty of Pharmacy, The British University in Egypt (BUE), El-Sherouk City, P.O. Box 43, Cairo 11837, Egypt; 4 Department of Virology and Immunology, Cancer Biology Department, National Cancer Institute, Cairo University, Kasr El-Aini, Cairo 11712, Egypt; 5 Department of Pharmacology and Toxicology, Faculty of Pharmacy, Cairo University, Kasr El-Aini, Cairo 11562, Egypt

## Abstract

Liver fibrosis is one of the most serious consequences of *S. mansoni* infection. The aim of the present study was to investigate the potential anti-fibrotic effect of human Wharton’s jelly-derived mesenchymal stem cells (WJMSCs) combined with praziquantel (PZQ) in *S. mansoni*-infected mice. *S. mansoni*-infected mice received early (8^th^ week post infection) and late (16^th^ week post infection) treatment with WJMSCs, alone and combined with oral PZQ. At the 10^th^ month post infection, livers were collected for subsequent flow cytometric, histopathological, morphometric, immunohistochemical, gene expression, and gelatin zymographic studies. After transplantation, WJMSCs differentiated into functioning liver-like cells as evidenced by their ability to express human hepatocyte-specific markers. Regression of *S. mansoni*-induced liver fibrosis was also observed in transplanted groups, as evidenced by histopathological, morphometric, and gelatin zymographic results besides decreased expression of three essential contributors to liver fibrosis in this particular model; alpha smooth muscle actin, collagen-I, and interleukin-13. PZQ additionally enhanced the beneficial effects observed in WJMSCs-treated groups. Our results suggest that combining WJMSCs to PZQ caused better enhancement in *S. mansoni*-induced liver fibrosis, compared to using each alone.

In hepatic schistosomiasis, the immunological response to the eggs laid down by adult worms in the liver tissue results in hepatic fibrosis, a direct sequel of the schistosomiasis-induced pathology^1^. Chemotherapy with praziquantel (PZQ) has remained the basis of schistosomiasis control for so long, owing to its broad spectrum, safety, and reasonable price^2^. Moreover, PZQ has proven efficacy against all *Schistosoma* species that infect humans with notable cure rates^3^. Additionally, the study of Liang *et al.*^4^ has reported potential alleviation of fibrosis caused by PZQ in advanced chronic schistosomiasis and CCl_4_-induced liver fibrosis where PZQ has decreased all the assessed fibrosis-related markers.

Stem cells and their possible use in cell therapy have drawn much attention recently, due to their potential for self-renewal and differentiation. For years, research has mainly focused on MSCs derived from bone marrow (BMSCs)^5^. Clinical application of BMSCs is, however, restrained by the aging of these cells as well as the invasive nature of the extracting procedure which compromises the isolated cells quantity and quality^6^. Many studies have emerged aiming at recognizing alternative sources of MSCs that can be obtained from a variety of sources without ethical controversies. The human umbilical cord Wharton’s jelly (WJ), the soft matrix that occurs within the umbilical cord surrounding the umbilical vessels, is considered a source of MSCs that are able to self-renew and differentiate into multiple cell types. Wharton’s jelly-derived MSCs (WJMSCs) may be superior to BMSCs for being more primitive representing an early-stage mesenchymal-like stem cells and more easily collected without ethical restrictions^5^. Additionally, previous studies have shown that WJMSCs minimally express major histocompatability complex class II antigens and costimulatory molecules suggesting that they are a favorable cell source for transplantation, without the need for immunosuppression^7^.

Thus, we hereby investigated the therapeutic potential of combining either early or late WJMSCs treatment to PZQ on both acute and chronic stages of *Schistosoma mansoni*-induced liver fibrosis in mice, respectively. Our results have suggested the capability of WJMSCs to differentiate into functioning liver-like cells. Using PZQ in combination with WJMSCs caused better enhancement in the *S. mansoni*-induced liver fibrosis, presumably by improving the microenvironment at sites of engraftment reaching for a better interaction between stem cells and the damaged liver tissue.

## Methods

### Animals

Swiss male albino mice CD-1, weighing 18–20 g each, were provided by the Schistosome Biology Supply Center (SBSC), Theodor Bilharz Research Institute (TBRI), Giza, Egypt. They were bred on a standard diet with free accessibility to water. The animals were kept under standard conditions of temperature (25 ± 0.5 °C), relative humidity (55 ± 1%) and light cycle (12 h light and 12 h dark). Moreover, all animal work was conducted in accordance with the guidelines outlined in the *Guide for the Care and Use of Laboratory Animals* and was approved by the Ethics Committee of the Faculty of Pharmacy, Cairo University (License no. PT 399).

### Infection

An Egyptian strain of *S. mansoni* cercariae was provided by the SBSC of TBRI. Cercariae were shed from laboratory bred infected snails namely, *Biomphalaria alexandrina*, 25–30 days after exposure to miracidia according to the method described by Pellegrino *et al.*^8^. Infection was done by subcutaneous (s.c.) injection of mice with 60 ± 10 *S. mansoni* cercariae suspended in 0.2 ml solution^9^.

### Isolation and culture of WJMSCs

The WJMSCs used in this study were originally isolated and expanded from a donated human umbilical cord with the donor’s consent after a full-term caesarean delivery, according to the method described by Salehinejad *et al.*^10^. The cells obtained were cultured in complete culture medium composed of low glucose Dulbecco’s Modified Eagle Medium (DMEM; Sigma-Aldrich Co., USA) with 2 mM L-glutamine, supplemented with 20% fetal bovine serum (FBS; Sigma-Aldrich Co., USA), and 100 U/L penicillin-streptomycin (Invitrogen, USA).

### Detection of WJMSCs surface markers

After expansion of cells through stepwise passaging, they were analysed for surface cell markers by flow cytometry as described previously^11^. Cells were incubated with the following anti-human antibodies, which were conjugated with fluorescein isothiocyanate (FITC) or phycoerythrin (PE) as follows: CD29–PE, CD44–FITC, CD59–PE, CD34–PE, CD105–PE, CD73–PE, CD90–PE, CD45–PE, CD14–FITC, CD19–PE, and HLA-DR–FITC. PE-conjugated IgG1 and FITC-conjugated IgG2a were used as isotype controls. Detection of PE and FITC labeling was accomplished using a FACScan flow cytometer (Beckman Coulter, USA).

### Transplantation of WJMSCs

Transplantation of WJMSCs into *S. mansoni*-infected mice was done by single intra-hepatic injection using 1.5 × 10^6^ cells/mouse suspended in DMEM^12^. The abdominal fur of each mouse was shaved with an electric fur shaver. The shaved abdominal skin was sterilized with a gauze swab that is moistened with a ready-to-use alcoholic solution for pre-operative treatment of the skin. The upper right quadrant area just below the liver was defined and marked in each mouse. A fractionated dose was injected directly into this area over a 15-min time to avoid sudden death of animals.

### Drugs and doses

Praziquantel (E.I.P.I.Co. Pharmaceuticals, Cairo, Egypt) was prepared as suspension in Cremophor-El and given orally seven weeks post infection at a dose of 500 mg/kg/day for two consecutive days^13^.

### Experimental design

Infected mice were randomly allocated into the following groups, each consisted of 10 mice:

Group I: This group was given orally Cremophor-El and/or injected with DMEM to serve as the infected control group. Group II: This group received early WJMSCs treatment at the 8^th^ week post infection. Group III: This group received late WJMSCs at the 16^th^ week post infection. Group IV: This group was treated with PZQ. Group V: This group received both early WJMSCs treatment (at the 8^th^ week post infection) and PZQ treatment. Group VI: This group received both late WJMSCs treatment (at the 16^th^ week post infection) and PZQ treatment. Sacrification of all mice was performed 10 months post infection.

Mortality percentages among control and treated groups were as follows: 20% in group I, 10% in group II, 20% in group III, 10% in group IV, 10% in group V, and 20% in group VI.

### Histopathological studies

Excised livers were immediately fixed in 10% formalin solution and embedded in paraffin. They were then processed and stained with hematoxylin and eosin (H&E) to examine the histopathological changes and with masson trichome to measure the mean granuloma diameter (μm) using an ocular micrometer (Zeiss, Germany), according to the method described by von Lichtenberg^14^.

### Morphometric Studies

Hepatic sections, 20 μm in thickness, were prepared and stained with Sirius red (SR) for the quantitation of collagen content using computer-controlled image analysis system (Leica, USA) as described previously^15^. Image analysis was performed using the computer software program, KS 200.

### Immunohistochemical studies

Immunohistochemistry was performed by using an avidin-biotin complex immunoperoxidase technique^16^ with anti-human primary antibodies against alpha fetoprotein (AFP) (Cat. No. sc-51506, Santa Cruz Biotechnology, CA, USA) cytokeratin (CK)-7 (Cat. No. sc-17116, Santa Cruz Biotechnology, CA, USA), CK-18 (Cat. No. sc-6259, Santa Cruz Biotechnology, CA, USA), Hep par-1 (Cat. No. sc-58693, Santa Cruz Biotechnology, CA, USA), oval cell marker type 6 (OV-6) (Cat. No. MAB2020, R&D Systems, MN, USA), alpha smooth muscle actin (α-SMA) (Cat. No. M0851, Dako, CA, USA), and vimentin (Cat. No. sc-53464, Santa Cruz Biotechnology, CA, USA) diluted at 1:100, in PBS. We used a streptavidin-biotin-peroxidase preformed complex and peroxidase-DAB (3,3′-diaminobenzidine) (Dako, Denmark), according to the manufacturer’s instructions. Sections were counterstained with Mayer’s hematoxylin and mounted with DPX medium. Positive and negative control slides for each marker were included in each session. As a negative control, a liver tissue section was processed as described, but with the primary antibody omitted.

### Gene expression analysis

Gene expression analysis was performed using quantitative real time polymerase chain reaction (qPCR). Total RNA was extracted and purified from liver tissue using RNeasy kit (Qiagen, USA) according to the manufacturer’s instructions. Complementary DNA was synthesized from RNA by reverse transcription (RT) using QuantiTect RT kit (Qiagen, USA). Human (h) and murine (m) primers used and their sequences are listed in Table 1. Expression of mRNA was assessed by qPCR using QuantiTect SYBR green PCR kit (Qiagen, USA) and ABI PRISM 7500 sequence detector (Applied Biosystems, CA, USA). Quantification was performed using the comparative Cq method, as described by Livak and Schmittgen^17^, normalized with the reference gene, β-actin.

### Gelatin zymography

Excised livers from sacrificed mice, previously stored at −80 °C were used. Liver tissue samples (50 mg) were weighed and homogenized in saline (0.9% NaCl solution) in a ratio of 1:4 w/v. The samples were centrifuged at 6,500 rpm and 4 °C then the supernatant which contained the matrix metalloproteinases (MMPs) was collected. Specific MMP-2 and MMP-9 activities were detected by gelatin zymography^18^ performed on premade 10% polyacrylamide gels containing 0.1% gelatin according to the instruction provided by manufacturer (Invitrogen, USA). On each gel, recombinant MMP-2 and MMP-9 (Merck Millipore, USA) were loaded as standards according to the manufacturer’s specifications. The bands were visualized by staining for 30 min with a solution containing 0.1% Coomassie R-250 (Sigma Aldrich, USA) in 40% ethanol and 10% acetic acid, followed by destaining for 2 h at room temperature in a solution containing 10% ethanol and 7.5% acetic acid. The relative activities of MMP-2 and MMP-9 for each sample were quantified by NIH ImageJ 1.42q software.

### Statistical analysis

All values are presented as means ± standard error (S.E.). Statistical analysis was performed by one-way analysis of variance (One-way ANOVA) followed by Bonferroni post hoc test for multiple comparisons using GraphPad Prism (v5). To test for an interaction between individual treatments when given in combination, a factorial design test was used^19^. In the absence of a significant interaction, the main effects were considered to be additive when combined. Statistical significance was determined at P < 0.05.

## Results

### Flow cytometry

**Figure 1:**
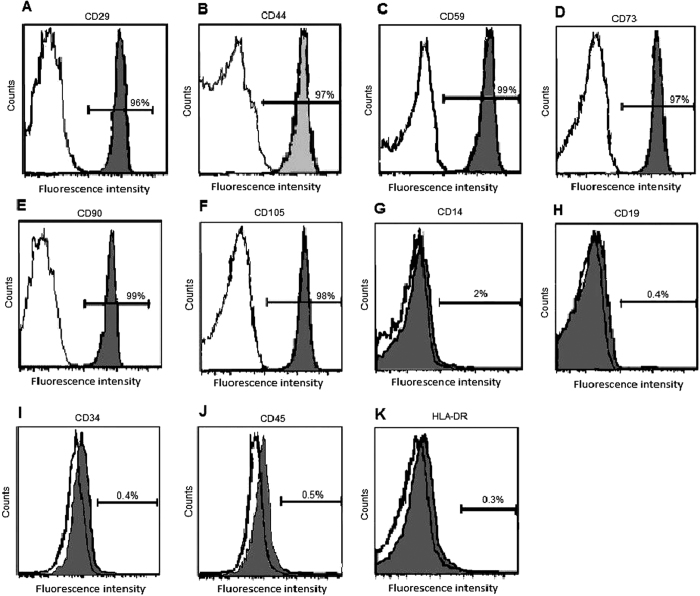
Immunophenotype of WJ-derived cells. Cells were labeled with FITC- or PE-conjugated antibodies. Shaded areas show the expression of (**A**) CD29, (**B**) CD44, (**C**) CD59, (**D**) CD73, (**E**) CD90, (**F**) CD105, (**G**) CD14, (**H**) CD19, (**I**) CD34, (**J**) CD45, and (**K**) HLA-DR.

Flow cytometric analysis showed that WJ-derived cells were positive for MSCs markers; CD29 (96%), CD44 (97%), CD59 (99%), CD73 (97%), CD90 (99%), and CD105 (98%) and negative for CD14 (2%), CD19 (0.4%), CD34 (0.4%), CD45 (0.5%), and HLA-DR molecules (0.3%) (Fig. 1).

### Histopathological studies

**Figure 2:**
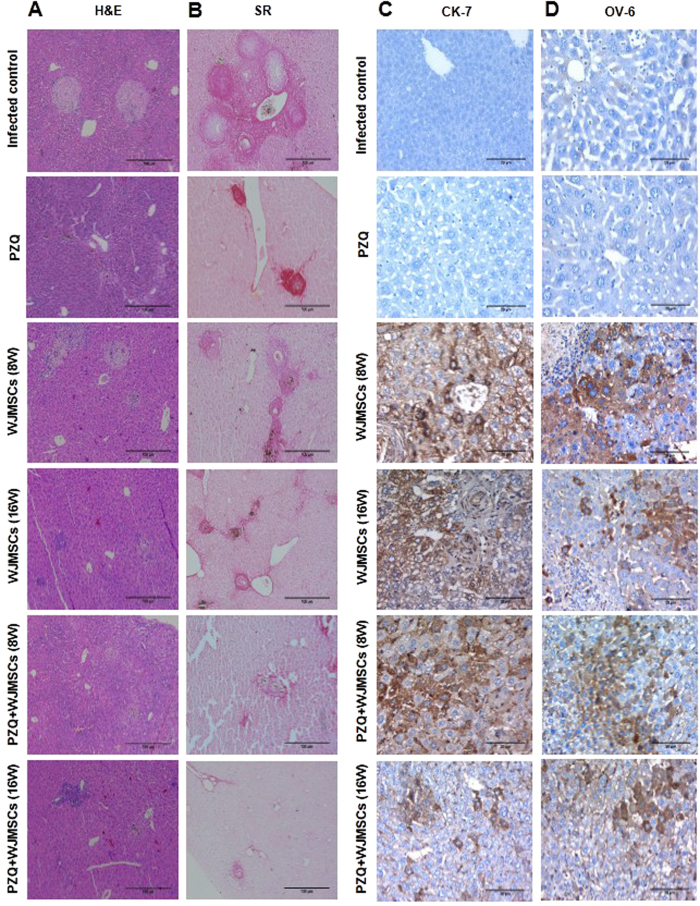
Photomicrographs of (**A**) haematoxylin and eosin (H&E, X100) and (**B**) Sirius red staining (SR, X100), as well as immunohistochemical staining (immunostain, DAB, X400) of (**C**) cytokeratin-7 (CK-7) and (**D**) oval cell marker type 6 (OV-6) of liver sections of control and treated groups. In the H&E- and SR-stained sections, fibrosis was less notable in the groups which received WJMSCs combined with PZQ where a reduction in granuloma diameters was observed. The fibrotic areas stained with Sirius red was significantly less in the groups which received combined treatment. As for the immunostained sections, no expression was observed in infected control and PZQ-treated groups. However, positive expression (brown cytoplasmic discolouration) was observed in all transplanted groups.

Histopathological examination of hepatic sections of *S. mansoni*-infected mice treated with PZQ showed small fibrocellular granulomas formed of degenerated ova surrounded by lymphocytes, giant cells, pigmented macrophages, plasma cells, and fibrous tissue (Fig. 2A). Moreover, the drug caused a decrease in the mean granuloma diameter by 47.49%, as compared to the infected control group (Table 2). Likewise, intra-hepatic treatment of infected mice with WJMSCs, at the 8^th^ and 16^th^ weeks post infection, showed a regression of the granulomatous inflammatory reaction resulting in a decrease in the mean granuloma diameter by 34.97 and 38.26%, as compared to the infected control group, respectively. In addition, combining either early or late WJMSCs treatment to PZQ resulted in a much higher regression of the inflammatory reaction around the bilharzial ova resulting in smaller fibrocellular granulomas, as compared to PZQ- and WJMSCs-treated groups. Both early and late WJMSCs combined with PZQ succeeded to reduce the mean granuloma diameters by 41.55 and 35.02%, as compared to PZQ-treated group, respectively (Table 2). The effect of the combination, however, was significantly less than additive.

### Morphometric studies

Morphometric analysis (Table 2) using SR staining (Fig. 2B) showed significant reductions in the percentage of the assessed fibrotic areas by 75.90% following PZQ treatment and by 63.04 and 62.29% following early and late WJMSCs treatment, respectively, as compared to the infected control group. Combining either early or late WJMSCs to PZQ caused reductions in the fibrotic areas by 58.04 and 51.77%, respectively, as compared to PZQ-treated group. The effect of the combination, however, was significantly less than additive.

### Immunohistochemical studies

**Figure 3:**
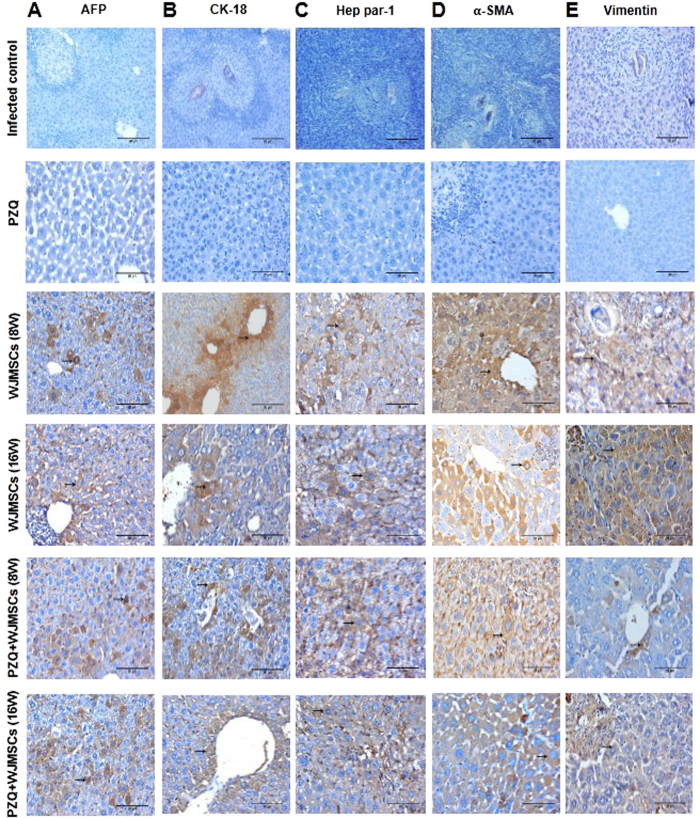
Photomicrographs of immunohistochemical staining (immunostain, DAB, X400) of (**A**) human alpha fetoprotein (AFP), (**B**) cytokeratin-18 (CK-18), (**C**) Hep par-1, (**D**) alpha smooth muscle actin (α-SMA), and (**E**) vimentin in liver sections of control and treated groups. No expression was observed in infected control and praziquantel (PZQ)-treated groups, whereas newly-formed liver-like cells in groups treated with Wharton’s jelly-derived mesenchymal stem cells (WJMSCs) showed positive expression (brown cytoplasmic discolouration) for AFP, CK-18, and Hep par-1. Positive expression of α-SMA and vimentin was observed only in scattered liver-like cells in groups treated with WJMSCs with the least expression observed in the groups which received WJMSCs combined with PZQ.

**Figure 4:**
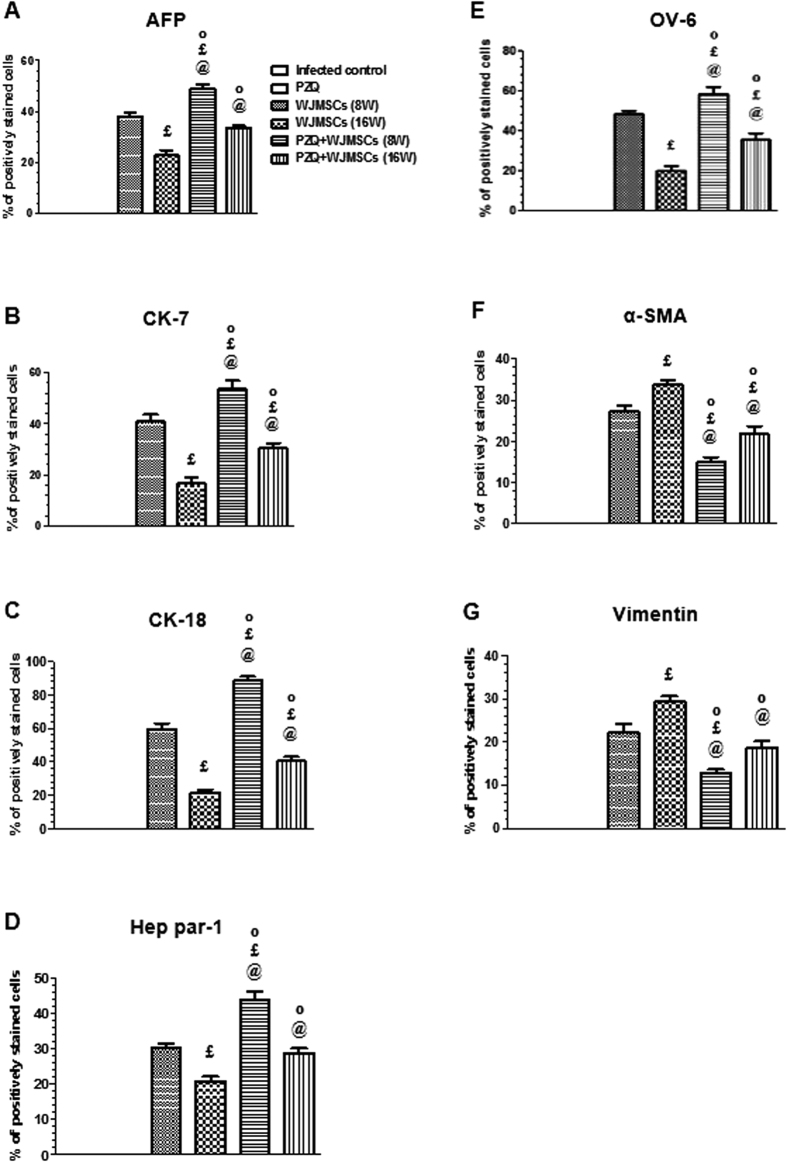
Effects of treatment with Wharton’s jelly-derived mesenchymal stem cells (WJMSCs), given either alone or combined with praziquantel (PZQ), on the immunohistochemical expression of human (**A**) alpha fetoprotein (AFP), (**B**) cytokeratin-7 (CK-7), (**C**) CK-18, (**D**) Hep par-1, (**E**) oval cell marker type-6 (OV-6), (**F**) alpha smooth muscle actin (α-SMA), and (**G**) vimentin in the liver sections of mice infected with *S. mansoni*. WJMSCs (1.5 × 10^6^ cells/mouse) were injected at either the 8^th^ (early) or 16^th^ (late) week (W) post infection. PZQ (500 mg/kg/day) was orally given at the 7^th^ W post infection for 2 consecutive days. Animals were sacrificed at the 10^th^ month post infection. Values are presented as means ± S.E. (n = 8–10). Significantly different (P < 0.05) ^£^ versus WJMSCs (8W), ^@^ versus WJMSCs (16W), and ^†^ versus PZQ + WJMSCs (8W). Statistical analysis was performed by one-way analysis of variance (One-way ANOVA) followed by Bonferroni post hoc test. ^O^ Significant interaction when PZQ and WJMSCs were combined using factorial design.

Human-specific antibodies with no cross-reactivity to mouse antigens were used to label the liver-associated markers, namely, AFP, CK-7, CK-18, Hep par-1, and OV-6, as well as MSC markers, namely, α-SMA and vimentin. The hepatic expression of AFP, CK-7, CK-18, Hep par-1, and OV-6 in the group which received late treatment with WJMSCs was reduced by 40, 58.74, 63.49, 31.33, and 58.59%, respectively, as compared to the group which received early treatment. Combining PZQ to early WJMSCs treatment caused 1.29-, 1.31-, 1.49-, 1.46-, and 1.21-fold higher expression of AFP, CK-7, CK-18, Hep par-1, and OV-6, respectively, as compared to the corresponding group which received WJMSCs treatment alone. Likewise, PZQ combined to late WJMSCs treatment caused 1.48-, 1.81-, 1.86-, 1.39-, and 1.79-fold higher expression of AFP, CK-7, CK-18, Hep par-1, and OV-6, respectively, as compared to the corresponding group which received WJMSCs treatment alone (Fig. 2C,D, Fig. 3A–C, and Fig. 4A–E).

The hepatic expression of human-specific α-SMA and vimentin in liver sections of mice infected with *S. mansoni* was further investigated. The group which received early treatment with WJMSCs showed reductions in α-SMA and vimentin hepatic expressions reaching 18.99 and 24.33%, as compared to the group which received late treatment. Combining PZQ to early WJMSCs caused 45.42 and 42.79% reductions in the expression of α-SMA and vimentin, respectively, as compared to the corresponding group which received WJMSCs treatment alone. Moreover, PZQ combined with late WJMSCs caused reductions in the hepatic expression of α-SMA and vimentin reaching 35.31 and 36.18%, respectively, as compared to the corresponding group which received WJMSCs treatment alone (Fig. 3D,E and Fig. 4F,G). As expected, there was no expression observed for all the aforementioned markers in the groups which are not treated with human-derived WJMSCs, viz., infected control and PZQ-treated groups (Fig. 3A–E). However, PZQ treatment significantly potentiated their hepatic expression when combined with WJMSCs treatment.

### Gene expression analysis

**Figure 5:**
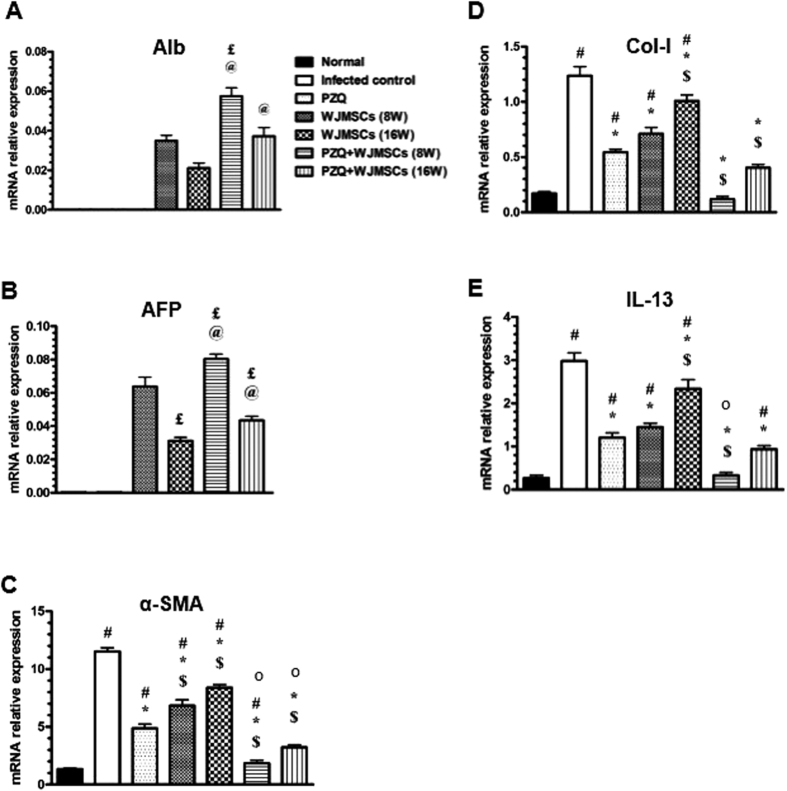
Effects of treatment with Wharton’s jelly-derived mesenchymal stem cells (WJMSCs), given either alone or combined with praziquantel (PZQ), on the gene expression of human (**A**) albumin (Alb) and (**B**) alpha fetoprotein (AFP) and murine (**C**) alpha smooth muscle actin (α-SMA), (**D**) collagen-I (Col-I), and (**E**) interleukin-13 (IL-13) in the liver tissue of mice infected with *S. mansoni*. WJMSCs (1.5 × 10^6^ cells/mouse) were injected at either the 8^th^ (early) or 16^th^ (late) week (W) post infection. PZQ (500 mg/kg/day) was orally given at the 7^th^ W post infection for 2 consecutive days. Animals were sacrificed at the 10^th^ month post infection. Values are presented as means ± S.E. (n = 8-10). Significantly different (P < 0.05) ^*^ vs infected control, ^$^ vs PZQ, ^£^ vs WJMSCs (8W), ^@^ vs WJMSCs (16W), and ^†^ vs PZQ + WJMSCs (8W). Statistical analysis was performed by one-way analysis of variance (One-way ANOVA) followed by Bonferroni post hoc test. ^O^ Significant interaction when PZQ and WJMSCs were combined using factorial design.

qPCR was performed in order to estimate the relative mRNA expression of the following genes: human albumin (Alb) and AFP as well as murine α-SMA, collagen I (Col-I), and interleukin 13 (IL-13). Results showed that mRNA relative expression of Alb and AFP were reduced by 40 and 51.56%, respectively, in the group which received late treatment with WJMSCs compared to the group which received early treatment. Combining PZQ to early WJMSCs treatment caused 1.66- and 1.25-fold higher expression of Alb and AFP, as compared to the group which received early WJMSCs treatment only, respectively. Moreover, combining PZQ to late WJMSCs treatment caused 1.76- and 1.42-fold higher expression of Alb and AFP, respectively, as compared to the group which received late WJMSCs treatment only (Fig. 5A,B). Similar to immunohistochmical results, gene expression of human Alb and AFP was undetermined in infected control and PZQ-treated groups, as expected. However, PZQ obviously potentiated the relative gene expression of both markers when combined with either early or late WJMSCs treatment.

On the other hand, PZQ showed a 57.76% reduction in mRNA relative expression of α-SMA, as compared to the infected control group. In addition, early and late WJMSCs treatment showed reductions in mRNA relative expression of α-SMA by 40.76 and 27.15%, respectively, as compared to the infected control group. Combining PZQ to early and late WJMSCs treatment showed 62.01 and 33.68% reductions in mRNA relative expression of α-SMA, respectively, as compared to PZQ-treated group (Fig. 5 C). Combining PZQ to early WJMSCs treatment caused a synergistic effect on α-SMA mRNA relative expression. However, the effect was significantly less than additive when PZQ was combined with late WJMSCs treatment.

Regarding Col-I and IL-13, oral PZQ treatment showed a 56.45 and 59.73% reductions in their mRNA relative expressions, respectively, as compared to the infected control group. Furthermore, early and late WJMSCs treatments caused reductions in mRNA relative expression of Col-I by 42.74 and 18.55% and IL-13 by 51.34 and 21.48%, respectively, as compared to the infected control group. Combining either early or late WJMSCs to PZQ showed 77.78 and 25.93% reductions in mRNA relative expression of Col-I as well as 72.5 and 21.67% reductions in IL-13 expression, respectively, as compared to PZQ-treated group (Fig. 5D,E). The combination caused an additive effect on the mRNA relative gene expression of Col-I. An additive effect was also observed on the mRNA relative expression of IL-13 when PZQ was combined with late WJMSCs treatment. Nevertheless, the effect was found to be significantly less than additive when PZQ was combined with early WJMSCs.

### Gelatin zymography

**Figure 6:**
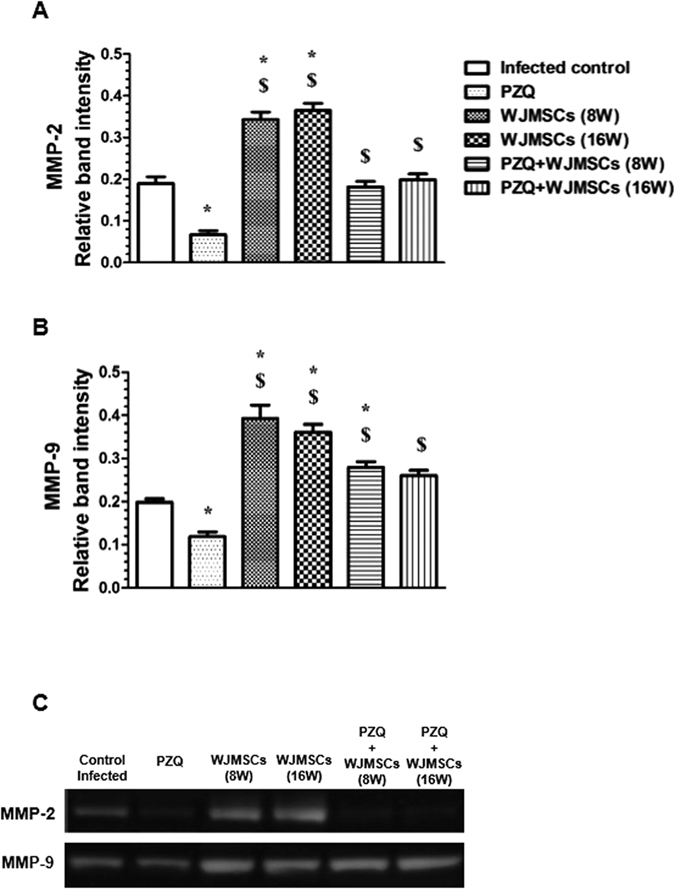
Effects of treatment with Wharton’s jelly-derived mesenchymal stem cells (WJMSCs), given either alone or combined with praziquantel (PZQ), on gelatin zymography of matrix metalloproteinase (MMP)-2 and -9 in the liver tissue of mice infected with *S. mansoni*. Quantitative analysis of the average relative band areas of (**A**) MMP-2 and (**B**) MMP-9. (**C**) Representative gelatin zymography showing the effects of different treatments on MMP-2 and -9 enzyme activities. WJMSCs (1.5x10^6^ cells/mouse) were injected at either the 8^th^ (early) or 16^th^ (late) week (W) post infection. PZQ (500 mg/kg/day) was orally given at the 7^th^ W post infection for 2 consecutive days. Animals were sacrificed at the 10^th^ month post infection. Values are presented as means ± S.E. (n = 8–10). Significantly different (P < 0.05) ^*^ vs infected control, and ^$^ vs PZQ. Statistical analysis was performed by one-way analysis of variance (One-way ANOVA) followed by Bonferroni post hoc test.

As shown in Fig. 6, oral PZQ caused a reduction in the estimated MMP-2 and MMP-9 average relative band areas by 64.74 and 40.20%, as compared to the infected control group, respectively. Additionally, Early and late WJMSCs treatments caused 1.81- and 1.92-fold increases in the measured MMP-2 average relative band areas, as compared to the infected control group, respectively. Moreover, the previously mentioned groups showed 1.98- and 1.82-fold increases in the measured MMP-9 relative band areas, respectively, as compared to the infected control group. Combining PZQ to either early or late WJMSCs treatment caused no change in the measured MMP-2 relative band areas, as compared to the infected control group. However, the measured MMP-9 average band area was increased by 1.40 fold in the group which received early combined treatment, as compared to the infected control group. Meanwhile, the average relative band areas of MMP-9 were statistically insignificantly increased by late combined treatment reaching 1.31 fold, as compared to the infected control group. Based on the statistical analysis performed, it was found that combining both treatments produced an additive effect on both MMP-2 and -9 enzyme activities.

## Discussion

Treatment and control of schistosomiasis still rely on the only available drug, PZQ, and hence the search for adjunctive therapies is compelling, particularly in view of rapid re-infection following treatment and emerging concerns about the potential development of tolerance and/or resistance to PZQ^20^. Currently, several clinical studies demonstrated the significance of stem cell-based therapies for the treatment of a wide range of human diseases. Recent studies^21^,^22^ had suggested that human WJMSCs are amongst the cord stem cell derivatives, the most promising one to generate functional human liver-like cells.

In this study, we provided data suggesting the differentiation of WJMSCs transplanted in *S. mansoni*-infected mice into functioning liver-like cells leading to regression of fibrosis-related markers, especially when PZQ was given in combination. The degree of fibrosis resolution was greatly dependant on the time at which treatment with WJMSCs was initiated. Hence, early treatment with WJMSCs has contributed more to the regression of fibrosis compared to later treatment. This could be attributed to the extent of collagen cross-linkage which consequently impacts its susceptibility to degradation by MMPs^23^.

After isolation of WJMSCs, the phenotypical screening showed that they expressed MSC-related antigens while negatively expressed haematopoietic antigens. These results are consistent with the criteria specified by the Mesenchymal and Tissue Stem Cell Committee of the International Society of Cellular Therapy (ISCT) to identify MSCs populations and to assure that they are not confounded by other cells^24^,^25^. Moreover, the differentiation of transplanted WJMSCs into functional liver-like cells was investigated by immunohistochemical analysis of human-specific AFP and CK-7 (early hepatocyte differentiation markers), as well as CK-18 and Hep par-1 (late hepatocyte differentiation markers) besides estimation of relative mRNA expression by qPCR of Alb (secretory plasma protein) and AFP^26^. Results of the previously mentioned studies indicated that WJMSCs differentiated into albumin-producing liver-like cells. These results are in agreement with previous studies^27^,^28^,^29^.

The cell compartment that resides in the canal of Hering has been called the progenitor (in humans) or the oval cell compartment (in rodents)^30^. The oval cells represent the progeny of hepatic stem cells and function as an amplification compartment for the generation of “new” hepatocytes^31^. In order to assess differentiation and to trace lineages of oval cells, a number of markers is commonly used which includes expressed antigenic markers for hepatocytes, biliary ducts and oval cells such as OV-6^32^, intermediate filaments, extracellular matrix proteins (CK-8, 18, 19), enzymes as well as secreted proteins (AFP and gamma-glutamyl transferase)^33^. In the present study, OV-6 was positively expressed in the hepatic tissue of *S. mansoni*-infected mice treated with either early or late WJMSCs.

Interestingly, the expression of the previously mentioned hepatic markers was increased in the groups which were treated with WJMSCs combined with PZQ. This may suggest that the surrounding environment may be essential for transplanted cells to differentiate into hepatocytes, which was at least partially enhanced in this study by PZQ treatment, as will be discussed later.

Treatment with WJMSCs, either alone or in combination with PZQ, had also succeeded to reduce mean granuloma diameters and the measured fibrotic areas. To help understand the previously mentioned findings, we performed gene expression analysis using qPCR, which showed a decline in the expression of three main contributors to hepatic fibrosis, particularly in this experimental model, namely, IL-13, Col-I, and α-SMA. IL-13 is a critical pro-fibrotic factor in *Schistosoma*-induced liver fibrosis since it was previously reported to suppress classical macrophage activation and have been implicated in granuloma formation and fibrosis around deposited eggs^34^,^35^. Furthermore, a recent study^36^ has shown that interfering with IL-13 pathway was capable of reducing the granulomatous area and *S. japonicum*-induced liver fibrosis in mice. Interestingly, previous studies have also demonstrated the ability of IL-13 to directly induce expression of Col-I and other critical fibrosis-associated genes, one of which is α-SMA, in hepatic stellate cells^37^,^38^.

According to previous studies, WJMSCs are capable of minimizing the deposition of collagen besides their ability to secrete hepatocyte growth factor which has an anti-apoptotic activity in hepatocytes and plays an essential role in liver regeneration, suggesting a beneficial effect for WJMSCs on the fibrotic process^28^. Accordingly, our results may suggest a potential protective role for WJMSCs in *S. mansoni*-induced liver fibrosis possibly by improving the liver microenvironment at sites of engraftment. This microenvironment may have been improved in presence of PZQ, since it was previously demonstrated that collagen synthesis subsides following eradication of worms, achieved in this study by PZQ, where subsequent reduction of egg deposition occurs. Consequently, the resolution of the inflammatory reaction at an early stage of *S. mansoni* infection could ultimately lead to reversal of fibrosis^39^. PZQ does not directly hold back collagen deposition in the liver^40^. Nevertheless, anti-inflammatory or immunomodulatory effects have been proposed^41^,^42^. PZQ, as previously reported, was also found to reduce IL-13 mRNA expression in *S. mansoni*-induced liver fibrosis^4^. The improvement of the fibrotic markers elicited by the combination treatment is in agreement with the study of Xu *et al.*^43^.

A longstanding controversy exists regarding the cellular origin of myofibroblasts in tissue fibrosis and whether MSCs contribute to fibrosis in multiple tissues^44^. Recent studies had suggested that MSCs are considered primary contributors to fibrogenesis in multiple organs^45^,^46^. It is verified that MSCs have intimate relationships with fibroblasts and that it is difficult to discriminate fibroblasts from MSCs based on phenotype (morphology and specific protein markers) or growth capacity^47^. Differentiated hepatocytes, however, gradually lose the expression of mesenchymal cell markers like α-SMA^48^. In our study, in order to confirm the mesodermal origin of WJMSCs and investigate whether they have contributed to fibrosis, the immunohistochemical expression of human-specific α-SMA and vimentin was measured. Our results showed that both markers were positively expressed in transplanted groups in scattered liver-like cells, however, their expression was greatly decreased when PZQ was administered in combination. This can be explained in light of the suggested anti-inflammatory and immunomodulatory effects of PZQ^41^,^42^, as previously mentioned, which may have optimized the conditions reaching for a better interaction between WJMSCs and the damaged liver environment.

The MMPs family plays an important role in collagen degradation^49^. Recently, the study of Lozito *et al.*^50^ has shown that human MSCs contained endogenous MMPs in both their cell-bound and conditioned media compartments suggesting that MSCs bind MMPs at their surfaces and that these MMPs were active. In addition, Wharton’s jelly was known to secrete certain MMPs^51^, which is supposed to help to degrade collagen in the fibrotic liver. Similarly, our results showed an increase in MMP-2 and -9 activities in transplanted mice, whereas a decrease in the measured enzyme activities was observed in PZQ-treated group. These results are in line with previous studies^28^,^52^.

In liver fibrosis, ECM components accumulate at about six times more than that in normal liver^53^. At an early stage of fibrosis, collagen types III & V besides fibronectin start to accumulate at the space of Disse^54^. At a later stage, increasing deposition of collagens types I & IV as well as other ECM components becomes more prominent^53^. Hence, early treatment of *S.mansoni*-induced liver fibrosis with WJMSCs (at the 8^th^ week post infection), while the ratio of collagen type III/I is normal, had led to decreased collagen deposition & hence significant anti-fibrotic effects. A less favorable, yet still beneficial, response was obtained when WJMSCs was given at a later stage post infection (at the 16^th^ week). This could be attributed to the decrease in the ratio of type III/I collagen with relative increase in type I collagen, which is very resistant to collagenases, owing to its triple helical structure^55^. Moreover, the varying activity of the ECM components and their remodelling enzymes, increased stiffness, and abundance of growth factors, are all considered very important determinants of the liver microenvironment at late stages of fibrosis compared to early stages that had presumably influenced the response to treatment, especially when PZQ was given in combination.

The use of MSCs in treating acute & chronic liver fibrosis has been recently demonstrated in a number of studies. However, our study aimed to target a number of specific mechanisms, which could explain the reported anti-fibrotic effects of WJMSCs combined with PZQ in *S. mansoni*-induced liver fibrosis. One of these mechanisms is the ability of the transplanted cells to transdifferentiate into intra-hepatic precursor cells (oval cells) as well as functioning liver-like cells where the results showed a marked ability of WJMSCs to express OV-6 as an oval cell marker as well as early and late differentiation markers which was found to be interestingly statistically potentiated in presence of PZQ. Also, the secretory function of those differentiated cells was found to be enhanced in presence of PZQ, as evidenced by the results of Alb mRNA expression. This mechanism might represent a possible explanation for the enhanced anti-fibrotic effects observed in the combination groups based on the significant potentiation exerted by PZQ. Another pathway, which was also investigated in the present study, is the potential immunomodulatory effect exerted by WJMSCs. This was fulfilled by examining, for the first time, the effect of WJMSCs on IL-13 in particular since it has been identified as the dominant effector cytokine in schistosomiasis-associated liver fibrogenesis^56^,^57^. The ability of MSCs to reduce IL-13 mRNA expression, even in absence of PZQ, could also explain the reduced granuloma diameter & fibrotic area as well as other subsequent anti-fibrotic effects such as the reduction in mRNA expression of Col-I and α-SMA (a key marker for hepatic stellate cell activation) observed in transplanted groups owing to the fact that IL-13 is the main cytokine known to regulate granuloma formation in this model. Thus, by reducing IL-13 levels, WJMSCs is suggested to have anti-inflammatory or anti-fibrotic effects in this model of liver fibrosis. Interestingly, the combination exerted an additive effect on both IL-13 & Col-I expression levels. Furthermore, the ability of early & late treatment with WJMSCs to enhance the activity of MMP-2 & −9 which are known for their collagenolytic activity could have partly contributed to the observed anti-fibrotic effects. This latter mechanism, however, cannot be considered to explain the overall alleviation of liver fibrosis exerted by the combination treatment since PZQ, when used in combination, have masked this beneficial effect by inhibiting the activity of both enzymes.

In conclusion, our results suggest that combining WJMSCs to PZQ enhanced the beneficial effects of PZQ on *S. mansoni*-induced liver fibrosis. Additionally, PZQ enhanced the differentiation capability of transplanted WJMSCs to functioning liver-like cells. In this study, we sought to specifically investigate human-derived WJMSC anti-fibrotic effects with the aim of applying these findings to clinical research. This is relevant since the isolation of murine WJMSC is challenging and obtaining homogenous populations compared to human cells is quite difficult. Additionally, localization & tracking of transplanted human-derived cells in the hepatic tissues of infected mice was much more applicable. In spite of the significant benefits emerging from MSC-based therapy, there are still safety issues to debate about, specifically regarding the long-term impact on immune function and the risk of malignancies which need to be addressed in future studies.

## Additional Information

**How to cite this article**: Hammam, O. A. *et al.* Wharton’s jelly-derived mesenchymal stem cells combined with praziquantel as a potential therapy for *Schistosoma mansoni*-induced liver fibrosis. *Sci. Rep.* **6**, 21005; doi: 10.1038/srep21005 (2016).

## Figures and Tables

**Table 1:** Sequence of human (h) and murine (m) primers used for quantitative real time polymerase chain reaction (qPCR) and their National Center for Biotechnology Information (NCBI) accession numbers.

Primer	Sequence	NCBI Accession number
**(h)Alb**	Forward: 5′- CCTTGGTGTTGATTGCCTTTGCTC -3′	**NM_000477**
	Reverse: 5′- CATCACATCAACCTCTGGTCTCACC -3′	
**(h)AFP**	Forward: 5′-TGAAATGACTCCAGTAAACCC-3′	**NM_001134**
	Reverse: 5′- AATGAGAAACTCTTGCTTCATC-3′	
**(m)α-SMA**	Forward: 5′- TGACCCAGATTATGTTTGA- 3′	**NM_007392**
	Reverse: 5′- GCTGTTATAGGTGGTTTCG- 3′	
**(m)Col-I**	Forward: 5′- CCTGGCAAAGACGGACTCAAC -3′	**NM_007742**
	Reverse: 5′- GCTGAAGTCATAACCGCCACTG -3′	
**(m)IL-13**	Forward: 5′- AGCCCTGGATTCCCTGAC- 3′	**NM_008355**
	Reverse: 5′- GCTGAGACCCTGAGCACTAG- 3′	
**(m)β-actin**	Forward: 5′- CTGTCCCTGTATGCCTCTG- 3′	**NM_007393**
	Reverse: 5′- ATGTCACGCACGATTTCC- 3′	

Abbreviations: Alb, Albumin; AFP, Alpha fetoprotein; α-SMA, Alpha smooth muscle actin; Col-I, Collagen-I; IL-13, Interleukin-13.

**Table 2:** Effects of treatment with Wharton’s jelly-derived mesenchymal stem cells (WJMSCs), given either alone or combined with praziquantel (PZQ), on the granuloma diameter and the fibrotic area (%) relative to the total area examined in Masson trichome- and Sirius red-stained hepatic sections of mice infected with *S. mansoni*, respectively.

Parameter	Infected control	PZQ	WJMSCs (8W)	WJMSCs (16W)	PZQ + WJMSCs (8W)	PZQ + WJMSCs (16W)
**Mean granuloma diameter**	173.30 ± 9.67	91.00 ± 4.06*	112.70 ± 5.60*	107.00 ± 5.90*	53.19 ± 3.81*$	59.13 ± 3.63*$
**Fibrotic area (%)**	10.58 ± 0.63	2.55 ± 0.11*	3.91 ± 0.29*$	3.99 ± 0.23*$	1.07 ± 0.13*$	1.23 ± 0.22*$

Mice were infected with 60 ± 10 cercariae/mouse, injected subcutaneously. WJMSCs (1.5 × 10^6^ cells/mouse) were once injected intra-hepatically at either the 8^th^ (early) or 16^th^ (late) week (W) post infection. PZQ (500 mg/kg/day) was orally given at the 7^th^ W post infection for 2 consecutive days. Animals were sacrificed at the 10^th^ month post infection. Data are represented as mean ± S.E. (n = 8–10). Significant difference (P < 0.05) * vs infected control, and ^$^ vs PZQ. Statistical analysis was performed by one-way analysis of variance (One-way ANOVA) followed by Bonferroni post hoc test.
