# Corrigendum: Wharton’s jelly-derived mesenchymal stem cells combined with praziquantel as a potential therapy for *Schistosoma mansoni*-induced liver fibrosis

**DOI:** 10.1038/srep32052

**Published:** 2016-09-02

**Authors:** Olfat A. Hammam, Nagwa Elkhafif, Yasmeen M. Attia, Mohamed T. Mansour, Mohamed M. Elmazar, Rania M. Abdelsalam, Sanaa A. Kenawy, Aiman S. El-Khatib

Scientific Reports 6: Article number: 2100510.1038/srep21005; published online: 02 15 2016; updated: 09 02 2016

In this Article, Affiliation 4 is incomplete. The correct affiliation is listed below:

Department of Virology and Immunology, Cancer Biology Department, National Cancer Institute, Cairo University, Children’s Cancer Hospital Egypt 57357, Cairo 11712, Egypt.

